# Tractable RNA–ligand interaction kinetics

**DOI:** 10.1186/s12859-017-1823-5

**Published:** 2017-10-16

**Authors:** Felix Kühnl, Peter F. Stadler, Sebastian Will

**Affiliations:** 1 Department of Computer Science and Interdisciplinary Center for Bioinformatics, University Leipzig, Härtelstr. 16-18, Leipzig, D-04107 Germany; 2 MPI for Mathematics in the Sciences, Inselstr. 22, Leipzig, D-04103 Germany; 3 FHI Cell Therapy and Immunology, Perlickstr. 1, Leipzig, D-04103 Germany; 4 Department Theoretical Chemistry, University Vienna, Währingerstr. 17, Wien, A-1090 Austria; 5 Bioinformatics and Computational Biology Research Group, Währingerstr. 17, Wien, A-1090 Austria; 6 RTH, University Copenhagen, Grønnegårdsvej 3, Frederiksberg C, 1870 Denmark; 7 Santa Fe Institute, 1399 Hyde Park Rd., Santa Fe, NM USA

**Keywords:** RNA secondary structure prediction, RNA interaction kinetics, RNA–ligand interaction, Riboswitches

## Abstract

**Background:**

The binding of small ligands to RNA elements can cause substantial changes in the RNA structure. This constitutes an important, fast-acting mechanism of ligand-controlled transcriptional and translational gene regulation implemented by a wide variety of riboswitches. The associated refolding processes often cannot be explained by thermodynamic effects alone. Instead, they are governed by the kinetics of RNA folding. While the computational analysis of RNA folding can make use of well-established models of the thermodynamics of RNA structures formation, RNA–RNA interaction, and RNA–ligand interaction, kinetic effects pose fundamentally more challenging problems due to the enormous size of the conformation space. The analysis of the combined process of ligand binding and structure formation even for small RNAs is plagued by intractably large state spaces. Moreover, the interaction is concentration-dependent and thus is intrinsically non-linear. This precludes the direct transfer of the strategies previously used for the analysis of RNA folding kinetics.

**Results:**

In our novel, computationally tractable approach to RNA–ligand kinetics, we overcome the two main difficulties by applying a gradient-based coarse graining to RNA–ligand systems and solving the process in a pseudo-first order approximation. The latter is well-justified for the most common case of ligand excess in RNA–ligand systems. We present the approach rigorously and discuss the parametrization of the model based on empirical data. The method supports the kinetic study of RNA–ligand systems, in particular at different ligand concentrations. As an example, we apply our approach to analyze the concentration dependence of the ligand response of the rationally designed, artificial theophylline riboswitch RS3.

**Conclusion:**

This work demonstrates the tractability of the computational analysis of RNA–ligand interaction. Naturally, the model will profit as more accurate measurements of folding and binding parameters become available. Due to this work, computational analysis is available to support tasks like the design of riboswitches; our analysis of RS3 suggests strong co-transcriptional effects for this riboswitch.

The method used in this study is available online, cf. Section “Availability of data and materials”.

## Background

Riboswitches enable the specific response to the presence of ligands by transcriptional or translational control of gene expression. Their ability to switch genes on or off depending on small molecules such as theophylline or tetracycline makes them valuable biotechnological tools. The design of tailored riboswitches for specific applications and advanced control logic is therefore an attractive endeavor in synthetic biology [1]. A riboswitch can be understood as the composition of its aptamer and its actuator domain. It senses the ligand by binding it to a binding pocket of the aptamer domain; this influences the conformations of the actuator domain and thereby leads to a measurable response to ligand binding, e. g. by terminating transcription (OFF-switch) or suppressing the terminator hairpin (ON-switch).

The computational design of artificial riboswitches requires a sufficiently accurate model of the ligand binding process and the structural response of the RNA to ligand binding. The equilibrium thermodynamics of RNA–ligand binding has been studied for RNA–RNA interactions, e. g. in [2, 3], and for small molecule binding in RNA–ligand [4]. As in the case of single molecule RNA folding, purely thermodynamic models are sometimes insufficient because they disregard the dynamics of the process. This can cause dramatic mis-predictions. Various approaches have analyzed the kinetics of single molecule RNA folding [5–8]. For tractability, the continuous process is decomposed into elementary steps, simplified based on heuristic assumptions, and/or approximated by a coarse-grained process.

Wolfinger et al. [7] present a coarse-graining approach to approximate RNA folding. Following e. g. [6], they analyze the folding process on the energy landscape of conformations, i. e. secondary structures, *R*_*i*_ of an RNA *R*. Conformation change is modeled by elementary moves (base pair insertion or deletion) endowed with reaction rates that follow the Arrhenius rule and thus depend on the energy barrier between the source and target conformations. In the approximation of RNA secondary structures, activation energies for opening/closing of single base pairs are approximately constant. The energy barrier thus effectively depends only on the energy difference between source and target [7]. This defines a Markov Process on the state space of all secondary structures, which is too large to make it possible to analyze it by diagonalizing the corresponding rate matrix. To effectively reduce the state space, [7] combine states into basins that consist of all conformations that are connected to the same local minimum by their gradient walk on the energy landscape. Since gradient walks connect states to their lowest energy neighbors, they correspond to the fastest folding paths from a state into a local minimum. This provides the rationale for approximating the full process by the *macroprocess* on gradient basin *macrostates*, which are assumed to be equilibrated. Consequently, the rates between the macrostates are canonically derived as weighted sums of *microrates* of the original process. By employing this heuristic approach, the size of the conformation space of smaller RNA molecules of up to a hundred nucleotides is typically reduced to just a few thousand macrostates. For example, in our analysis of the 81 nucleotide long riboswitch RS3 [1], 11.4 millions of secondary structures are mapped to 1133 macrostates. The macroprocess is finally solved by diagonalization. In our approach we re-use ideas of this coarse-graining, which also allows us to re-use several tools for single-molecule RNA kinetics (RNAsubopt [9], barriers [10], treekin [7]).

### RNA–ligand interaction model

We are going to describe a reaction system of the RNA *R* (given by its sequence of nucleotides {*A*,*C*,*G*,*U*}) with the ligand *L* at the level of RNA and binding complex conformations, such that we can study the kinetics of association, dissociation, and conformation changes. For simplicity, we assume that there is only a single ligand conformation (also denoted *L*). In the same way as a single RNA molecule transitions between various conformations during the folding process, the complex of RNA and ligand adopts different conformations *L* *R*_*i*_. Importantly, only a subset of the RNA conformations binds the ligand. The part of the total state space that corresponds to the RNA–ligand complexes is therefore isomorphic to a subset of the state space of the free RNA molecule. Thus, our system of consideration consists of the reactions

1 $$\begin{array}{*{20}l} R_{i} &\longrightarrow R_{j}  \end{array} $$

2 $$\begin{array}{*{20}l} L + R_{i} &\longrightarrow LR_{i}  \end{array} $$

3 $$\begin{array}{*{20}l} ~~~~~~LR_{i} &\longrightarrow L + R_{i}  \end{array} $$

4 $$\begin{array}{*{20}l} ~~~~~LR_{i} &\longrightarrow LR_{j}, \end{array} $$

for all *i*,*j*∈{1,…,*N*}, *i*≠*j*. According to the rate laws for elementary reactions, the rates of each of these reactions depend on specific rate constants and the concentrations of the molecules. The reactions 1 and 4 only have non-zero rate constants, if the RNA conformations *R*_*i*_ and *R*_*j*_ are related by an elementary move such as the insertion or deletion of a base pair. Moreover, *L* and *R*_*i*_ can interact only for the subset of the *R*_*i*_ that form an appropriate binding pocket; otherwise, the complex *L* *R*_*i*_ is deemed unstable and thus excluded from the model. Since RNA conformations correspond to RNA secondary structures, the energies of monomer states can be calculated from the Turner energy model [11]. For dimer states, we add the aptamer–ligand-specific binding energy. For the exemplary studied riboswitch RS3, this energy can be derived from the empirical dissociation constant [1]. Finally, we derive the rate constants as Metropolis rates with appropriate pre-exponential factors that can be estimated from empirical rates. Note that the rates of base pair opening *k* ^−^ and closing *k* ^+^ are directly related by the energy change *Δ* *G* due to the closing. Concretely, *k* ^−^/*k* ^+^= exp(*Δ* *G*/*R* *T*) for *Δ* *G*<0. Experimental values are available for the zippering rate, which corresponds to the rate of closing the last hairpin in a helix. A careful analysis in [12] yields a value in the range 4.7·10^7^ to 10^9^s^−1^ roughly consistent with earlier estimates [13–15]. In principle, a kinetic constant can be derived for the closing of first base pair in a loop from a worm-like chain model [12, 16]; following earlier work on RNA kinetic models [7], we use here a single kinetic parameter *k* ^+^ for all base pairs. An empirical rate of one specific theophylline aptamer association was reported as 600 M^−1^s^−1^ [17], which may serve as rough estimate for comparable systems. Note that [17] measured the macroscopic *apparent rate* that depends on the rate of dimerization as well as the rate of refolding into structures with theophylline binding pocket.

While the Reactions 1, 3, and 4 are of first order, the second order association in Reaction 2 introduces non-linearity into the system. Assuming ligand excess, which is a very plausible assumption for small molecular ligands, however, it is possible to devise a *pseudo-first order approximation* of the system.

**Fig. 1:**
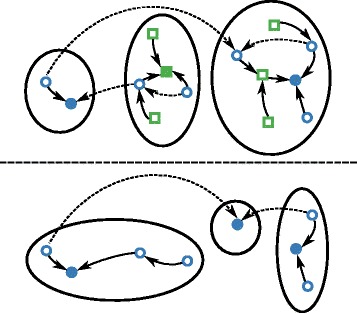
Relation between the monomer energy landscape *Ξ* (*above*) and the dimer energy landscape *Ξ* ^∗^ (*below*). We obtain the landscape of the dimers from the landscape of the monomers by constraining the structures to contain the binding pocket. *Blue circles* indicate structures with binding pocket, while the remaining structures are shown as *green squares*. Notably, the assignment to gradient-basins regularly differs for corresponding structures in both landscapes, if gradient neighbors (*solid arrow* transitions) of the monomer world have no binding pocket such that non-gradient neighbors (*dashed arrow* transitions) of the monomer world correspond to gradient neighbors in the dimer world. Filled circles and squares mark local minima

Even if the reaction equations above appropriately model the RNA–ligand interaction, this system is still computationally intractable for typical riboswitch sizes. As a remedy, we construct a coarse-grained process based on the separate gradient-basins for the monomer and dimer-states. The monomer states with suitable binding pocket are connected to dimer states, cf. Fig. 1. Importantly, there is no direct mapping from monomer macrostates to dimer macrostates of our coarse-grained system because conformations without binding pockets are absent from the “dimer world”. The upper basin in the “monomer world” of Fig. 1 is subdivided into two basins in the dimer world; conversely, the middle and lower monomer basins correspond to a single basin of the dimer world.

### Contributions

We start by elaborating the general macroprocess of RNA–ligand interaction, based on gradient basin macrostates, and derive the corresponding rate constants. This contributes an original description of coarse-grained interaction processes; it is the first fundamental prerequisite for our tractable RNA–ligand kinetics approach. Furthermore, we leverage that a wide spectrum of biological RNA–ligand systems operate under strong ligand excess, justifying the pseudo-first order approximation. On these grounds, we establish the first analytical approach for RNA–ligand interaction kinetics. Based on solving the master equation of the interaction process, this enables the computation of time-dependent macrostate probabilities. Finally, we study the kinetics of the artificially designed riboswitch RS3 [1] interacting with theophylline. We analyze the system at different concentrations and present results that strongly suggest co-transcriptional effects.

## Methods

We consider the fixed interaction system of the *RNA* *R* and the *ligand* *L*. Let *X* denote the set of all *monomer microstates*, *X*={*R*_*i*_∣*i*=1,…,*N*}; in our setting, the *R*_*i*_ are the secondary structures of a given RNA sequence. The subset *X* ^+^⊆*X* comprises the conformations that can bind the ligand. Here *X* ^+^ contains all states with a specific binding pocket. Furthermore, define *X* ^∗^ as the set of *dimer microstates* *L* *R*_*i*_, *X* ^∗^={*L* *R*_*i*_∣*R*_*i*_∈*X* ^+^}⊆{*L* *R*_*i*_∣*i*=1,…,*N*}.

A dimer microstate *L* *R*_*i*_∈*X* ^∗^ has the energy *E*(*R*_*i*_)+*θ*, where *θ*<0 denotes the *binding energy* of *R* and *L*. The *inverse temperature* is $\mathfrak {b}=\frac {1}{RT}$, where *T* is the *absolute temperature* and *R* is the *universal gas constant*. For a set *S*⊆*X*∪*X* ^∗^ of microstates, let $Z[\,{S}\,] := \sum _{x\in S}\exp (-\mathfrak {b} E(x))$ denote the partition function of *S*. The *probability of a microstate x* in *S* is then given by

$$\text{Pr}[\,x \mid S\,]:= \left\{\begin{array}{ll} \exp(-\mathfrak{b} E(x))\,/\,Z[\,{S}\,] & \text{if}\,\, x \in S, \\ 0 & \text{otherwise.} \end{array}\right. $$

Let *x*,*y*∈*X*∪*X* ^∗^ be microstates. The *microrate constant* from *x* to *y* is denoted *k*(*x*→*y*) (or *k*(*y*←*x*)). On microstates *x*,*y*, we define the symmetric *neighborhood relation* $\,\mathcal {N}{}$ such that ${x}\,\mathcal {N}{y}$ holds if and only if *x* and *y* have a distance of exactly one elementary move.

For ${x}\,\mathcal {N}{y}$, let

$$ E^{\Delta}_{xy} := \max\{E(x),E(y)\}-E(x) $$

be the activation energy of the transition *x*→*y* as defined by the Metropolis rule. Accordingly, the microrate constants for distinct states *x*,*y*∈*X*∪*X* ^∗^, where ${x}\,\mathcal {N}{y}$, are defined as

$$k({x \rightarrow y}):=A({x \rightarrow y}) \exp(-\mathfrak{b} E^{\Delta}_{xy}), $$

otherwise, define *k*(*x*→*y*):=0. *A*(*x*→*y*) denotes the reaction-specific *pre-exponential factor*. For our purposes, we assume that this factor depends only on the type of reaction and the factors for conformation change in monomers and dimers are equal. Thus, we distinguish the factors *A*_a_ for association, *A*_d_ for dissociation, and *A*_R_ for conformation changes of the RNA secondary structure. As we will show later, *A*_a_=*A*_d_ due to detailed balance.

Denote the *powerset* of a set *S* by $\operatorname {\mathcal {P}}(S)$. A *monomer (or dimer) macrostate* is a set of monomer (or dimer) microstates, i. e. an element of $\operatorname {\mathcal {P}}(X)$ ($\operatorname {\mathcal {P}}({X}^{*}))$. We denote the *(macro)rate* constant from macrostate *α* to *β* by *r*(*α*→*β*) (or *r*(*β*←*α*)). Macrorate constants are defined by summing over microrate constants and their respective state probabilities, i. e.

$$r({\alpha\rightarrow\beta}) := \sum_{{x\in\alpha,y\in\beta}} \text{Pr}[\,x\mid\alpha\,] \cdot k\left({x \rightarrow y}\right). $$

We emphasize that we use the term macrostates freely to denote general sets of microstates. Only when we introduce specific partitions of the microstates into macrostates, it makes sense to distinguish *represented* macrostates of our specific coarse-grained system from other sets of microstates.

## Results and discussion

### Macrostate kinetics of RNA–ligand interaction

For a microstate *x*∈*X* ^+^, we denote its corresponding dimer microstate (after binding to *L*) by *Lx*, i. e. for *x*=*R*_*i*_, *L* *x*=*L* *R*_*i*_. This notation is raised to sets of microstates by defining *L* *α*:={*L* *x*∣*x*∈*α*}. Lemma 1 below asserts that the rate constants between dimer microstates and macrostates can be computed exactly like rate constants of monomer states.

#### **Lemma 1**

For *x*,*y*∈*X* ^+^, *k*(*L* *x*→*L* *y*)=*k*(*x*→*y*). Furthermore, Pr[ *L* *x*∣*L* *α* ]=Pr[ *x*∣*α* ] holds for all $\alpha \in \operatorname {\mathcal {P}}({X}^{+})$. Finally, *r*(*L* *α* → *L* *β*)=*r*(*α*→*β*), for all macrostates $\alpha,\beta \in \operatorname {\mathcal {P}}({X}^{+}).$

#### *Proof*

The individual claims follow easily from the definitions. If $({x},{y})\notin \mathcal {N}$, *k*(*x*→*y*)=0=*k*(*L* *x*→*L* *y*), so assume ${x}\,\mathcal {N}{y}$. Since

$$ {\displaystyle \begin{array}{lll}{E}_{LxLy}^{\varDelta }& =\max \left\{E(Lx),E(Ly)\right\}-E(Lx)\kern2em & \kern2em \\ {}& =\max \left\{E(x)+{\theta}_L,E(y)+{\theta}_L\right\}\kern2em & \kern2em \\ {}& \kern2em -\left(E(x)+{\theta}_L\right)\kern2em & \kern2em \\ {}& =\max \left\{E(x),E(y)\right\}+{\theta}_L\kern2em & \kern2em \\ {}& \kern2em -E(x)-{\theta}_L\kern2em & \kern2em \\ {}& ={E}_{xy}^{\varDelta}\kern2em \\ {\mathrm{holds},}\\ {}\kern2em \\ {}k\left( Lx\to Ly\right)& =A\left( Lx\to Ly\right)\exp \left(-\mathfrak{b}\underset{LxLy}{\overset{\varDelta }{E}}\right)\kern2em & \kern2em \\ {}& =A\left(x\to y\right)\exp \left(-\mathfrak{b}\underset{xy}{\overset{\varDelta }{E}}\right)\kern2em & \kern2em \\ {}& =k\left(x\to y\right).\kern2em & \kern2em \end{array}} $$

Furthermore,

$$\begin{array}{*{20}l} \text{Pr}[\,Lx\mid L\alpha\,] &= \frac{\exp(-\mathfrak{b} E(Lx))}{\operatorname{Z}[\,{L\alpha}\,]} \\ &= \frac{\exp(-\mathfrak{b} [E(x)+\theta_{L}])} {\sum_{x\in\alpha} \exp(-\mathfrak{b} [E(x)+\theta_{L}])} \\ &= \frac{\exp(-\mathfrak{b} \theta_{L})\exp(-\mathfrak{b} E(x))} {\exp(-\mathfrak{b} \theta_{L}) \sum_{x\in\alpha} \exp(-\mathfrak{b} E(x))} \\ &= \text{Pr}[\,{x}\mid\alpha\,]. \end{array} $$

Finally,

$$\begin{array}{*{20}l} r({L\alpha{\rightarrow}\,{L}\beta}) &= \sum_{{\substack{Lx\in L\alpha\\Ly\in L\beta}}} \text{Pr}[\,Lx\mid L\alpha\,] \cdot k({Lx \rightarrow Ly}) \\ &= \sum_{{\substack{x\in\alpha\\y\in\beta}}} \text{Pr}[\,x\mid \alpha\,] \cdot k({x \rightarrow y}) \\ &= r({\alpha\rightarrow\beta}). \end{array} $$

□

The microrate constant from monomer to dimer states is constant, whereas the back rate depends on the binding energy *θ*.

#### **Lemma 2**

(Association and dissociation microrate constants) For *x*∈*X* ^+^, the rate of association is *k*(*x*→*L* *x*)=*A*_a_, while the dissociation rate is $k({Lx \rightarrow x})=A_{\mathrm {d}}\exp (\mathfrak {b} \theta).$ All other rates between monomer and dimer microstates are *0*.

#### *Proof*

By Metropolis rule, for *x*∈*X* ^+^,

$$\begin{array}{*{20}l} k({x \rightarrow Lx}) &= A_{\mathrm{a}}\exp\left(-\mathfrak{b} E^{\Delta}_{xLx}\right) \\ &= A_{\mathrm{a}}\exp\left(-\mathfrak{b}[E(x)-E(x)]\right) \\ &= A_{\mathrm{a}}, \end{array} $$

since $E^{\Delta }_{xLx} = \max \{E(x),E(Lx)\}-E(x)$ and, additionally, *E*(*L* *x*)=*E*(*x*)+*θ*_*L*_≤*E*(*x*). Analogously, for the inverse microrate,

$$\begin{array}{*{20}l} k({Lx \rightarrow x}) &= A_{\mathrm{d}}\exp\left(-\mathfrak{b} E^{\Delta}_{xLx}\right) \\ &= A_{\mathrm{d}}\exp(-\mathfrak{b}[E(x)-E(Lx)]) \\ &= A_{\mathrm{d}} \exp(\mathfrak{b}\theta_{L}). \end{array} $$

□

The association (dissociation) microrates due to Lemma 2 induce corresponding macrorates, which additionally depend on the probability of the associable (dissociable) microstates in the source macrostate, respectively (Lemma 3).

#### **Lemma 3**

(Association and dissociation macrorate constants) For arbitrary $\alpha \in \operatorname {\mathcal {P}}(X)$ and $\beta \in \operatorname {\mathcal {P}}({X}^{+})$, equation $r({\alpha {\rightarrow }\,L\beta })= A_{\mathrm {a}}\frac {Z[\,{\alpha \cap \beta }\,]}{Z[\,{\alpha }\,]} $ holds. Additionally, $r({L\beta \rightarrow \alpha })= A_{\mathrm {d}}\frac {Z[\,{\alpha \cap \beta }\,]}{Z[\,{\beta }\,]}\cdot \exp (\mathfrak {b}\theta).$

#### *Proof*

Let $\alpha \in \operatorname {\mathcal {P}}(X)$ and $\beta \in \operatorname {\mathcal {P}}(X^{+})$. Thus

$$ {\displaystyle \begin{array}{lll}r\left(\alpha \to \kern0.3em \mathrm{L}\upbeta \right)& =\sum \limits_{\begin{array}{c}x\in \alpha \\ {} Ly\in \mathrm{L}\upbeta \end{array}}\Pr \left[\kern0.3em x\mid \alpha \kern0.3em \right]\cdotp k\left(x\to Ly\right)\kern2em & \kern2em \\ {}& =\sum \limits_{x\in \alpha \cap \beta}\Pr \left[\kern0.3em x\mid \alpha \kern0.3em \right]\cdotp k\left(x\to Lx\right)\kern2em & \kern2em \\ {}& ={A}_{\mathrm{a}}\frac{\mathrm{Z}\left[\kern0.3em \alpha \cap \beta \kern0.3em \right]}{\mathrm{Z}\left[\kern0.3em \alpha \kern0.3em \right]}\kern2em \\ {}{\mathrm{a}\mathrm{nd}}\\ {}\kern2em \\ {}r\left(\mathrm{L}\upbeta \to \alpha \right)& =\sum \limits_{\begin{array}{c} Lx\in \mathrm{L}\upbeta \\ {}y\in \alpha \end{array}}\Pr \left[\kern0.3em Lx\mid \mathrm{L}\upbeta \kern0.3em \right]\cdotp k\left( Lx\to y\right)\kern2em & \kern2em \\ {}& =\sum \limits_{x\in \alpha \cap \beta}\Pr \left[\kern0.3em Lx\mid \mathrm{L}\upbeta \kern0.3em \right]\cdotp k\left( Lx\to x\right)\kern2em & \kern2em \\ {}& ={A}_{\mathrm{d}}\frac{\mathrm{Z}\left[\kern0.3em \alpha \cap \beta \kern0.3em \right]}{\mathrm{Z}\left[\kern0.3em \beta \kern0.3em \right]}\cdotp \exp \left(\mathfrak{b}{\theta}_L\right).\kern2em & \kern2em \end{array}} $$

□

### A tractable model under ligand excess

For our coarse-grained RNA–ligand interaction process, we partition the monomer microstates *X* and the dimer microstates *X* ^∗^ into sets of macrostates *Ξ* and *Ξ* ^∗^, respectively. For the theoretical discussion, we require only that *Ξ* and *Ξ* ^∗^ are partitions of the respective sets *X* and *X* ^∗^. Later, in our application, we are going to define macrostates as gradient basins (within their respective component).

We denote the monomer macrostates (in *Ξ*) by *α*_1_,…,*α*_*n*_ and the dimer macrostates (in *Ξ* ^∗^) by *β*_1_,…,*β*_*m*_. Since—by model assumption—the ligand is in large excess, the change of the ligand concentration [*L*] is essentially negligible in relation to the change of RNA concentrations. Formally, we assume *d*/*d* *t*[*L*]=0, i. e. at all times [*L*]=*l*_0_, for the initial ligand concentration *l*_0_. The change of RNA monomer and RNA–ligand dimer concentrations over time is described by a system of ordinary differential equations (ODEs) corresponding to Reactions (1)–(4).

Following the first-order rate laws, Reaction (1) causes *n* ^2^−*n* flows *r*(*α*_*i*_→*α*_*j*_)[*α*_*i*_] from *α*_*i*_ to *α*_*j*_ (1≤*i*,*j*≤*n*, *i*≠*j*); Reaction (4) *m* ^2^−*m* flows *r*(*β*_*i*_→*β*_*j*_)[*β*_*i*_] from *β*_*i*_ to *β*_*j*_ (1≤*i*,*j*≤*m*, *i*≠*j*); and Reaction (3) *n*·*m* flows *r*(*β*_*i*_→*α*_*j*_)[*β*_*i*_] from *β*_*i*_ to *α*_*j*_ (1≤*i*≤*m* and 1≤*j*≤*n*). In contrast to these simple first-order transitions, the state changes due to Reaction 2 follow second-order rate laws contributing the *n*·*m* flows *r*(*α*_*i*_→*β*_*j*_)[*L*][*α*_*i*_] from *α*_*i*_ to *β*_*j*_ (1≤*i*≤*n* and 1≤*j*≤*m*). Without the assumption *d*/*d* *t*[*L*]=0, the rate would depend on two variable concentrations, causing the system to be non-linear. However, by our assumption, the concentration [*L*] is constant.

**The system of ODEs.** The change of concentrations is now described by summing over single contributions:

$$\begin{array}{*{20}l} \frac{d}{dt} [\alpha_{i}] =&\phantom{+{}} \sum_{{\substack{1\leq k\leq n\\k\neq i}}} r({\alpha_{k}\rightarrow\alpha_{i}})[\alpha_{k}]\\ &+ \sum_{{1\leq k\leq m}} r({\beta_{k}\rightarrow\alpha_{i}})[\beta_{k}]\\ &- \sum_{{\substack{1\leq k\leq n\\k\neq i}}} r({\alpha_{i}\rightarrow\alpha_{k}})[\alpha_{i}]\\ &- \sum_{{1\leq k\leq m}} r({\alpha_{i}\rightarrow\beta_{k}})[L][\alpha_{i}] \end{array} $$

for *i*=1,…,*n*, and

$$\begin{array}{*{20}l} \frac{d}{dt} [\beta_{j}] =&\phantom{+{}} \sum_{{1\leq k\leq n}} r({\alpha_{k}\rightarrow\beta_{j}})[L][\alpha_{k}] \\ &+ \sum_{{\substack{1\leq k\leq m\\k\neq j}}} r({\beta_{k}\rightarrow\beta_{j}})[\beta_{k}] \\ &- \sum_{{1\leq k\leq n}} r({\beta_{j}\rightarrow\alpha_{k}})[\beta_{j}] \\ &- \sum_{{\substack{1\leq k\leq m\\k\neq j}}} r({\beta_{j}\rightarrow\beta_{k}})[\beta_{j}] \end{array} $$

for *j*=1,…,*m*.

We set *γ*:=(*α*_1_,…,*α*_*n*_,*β*_1_,…,*β*_*m*_)^*T*^ and define the (*n*+*m*) × (*n*+*m*)-matrix *R*(*l*_0_). Then the entire coarse-grained system under ligand excess can be expressed by the linear ODE $ \frac {d}{dt} [\gamma ] = R(l_{0}) [\gamma ], $ where

$$R(l_{0})= \left(\begin{array}{cc} A & \quad C \\ l_{0} \cdot D & \quad B \end{array} \right) $$

is constructed from four submatrices:

- is an *n* × *n*-matrix with entries *a*_*ij*_=*r*(*α*_*i*_←*α*_*j*_) for 1≤*i*,*j*≤*n*, *i*≠*j*. For 1≤*i*≤*n*,
  $$ a_{ii} := -\sum_{{\substack{1\leq k\leq n\\k \neq i}}} r({\alpha_{k} \leftarrow \alpha_{i}}) -\sum_{{1\leq k\leq m}} l_{0} r({\beta_{k} \leftarrow \alpha_{i}}). $$
- is an *m* × *m*-matrix with entries *b*_*ij*_=*r*(*β*_*i*_←*β*_*j*_) for 1≤*i*,*j*≤*m*, *i*≠*j*. For 1≤*j*≤*m*,
  $$ b_{jj} := -\sum_{{1\leq k\leq n}}r({\alpha_{k} \leftarrow \beta_{j}}) - \sum_{{\substack{1\leq k\leq m\\k \neq j}}} r({\beta_{k} \leftarrow \beta_{j}}). $$
- is an *n* × *m*-matrix with entries *c*_*ij*_=*r*(*α*_*i*_←*β*_*j*_) for 1≤*i*≤*n*,1≤*j*≤*m*, and
- is an *m* × *n*-matrix with entries *d*_*ij*_=*r*(*β*_*i*_←*α*_*j*_) for 1≤*i*≤*m*,1≤*j*≤*n*.

### Computing RNA–ligand kinetics

The described ODE system can be solved analytically building on existing software. The entire computation pipeline consists of five major steps:

1. enumeration of the RNA’s structure space
2. computation of the gradient basins and corresponding rates for
  - the monomer landscape
  - the dimer landscape
3. computation of the rates between the monomer and dimer basins
4. construction of the full rate matrix *R*(*l*_0_)
5. integration of the linear ODE system

Since an exhaustive enumeration of the structure space is infeasible even for short RNAs, *Step 1* generates only a selected part of all possible secondary structures of the input RNA. For this work, we consider only structures up to a certain energy above the minimum free energy of the sequence as computed by RNAsubopt [9]. To further reduce the number of structures, only structures without any isolated base pairs are generated.

Often, the restriction to low energy structures excludes important microstates of relatively high energy such as the open RNA chain from the model. Simply adding such a structure to the system is insufficient without also including transitional structures that connect it to the remaining states. The solution for this work was to develop a heuristic algorithm to partially explore an energy landscape around a given structure of interest, flooding neighbored basins if their local minimum has a lower energy than the current one, until one reaches structures within the already explored energy band. This approach seems to be more adequate in the context of a gradient basin coarse graining than a direct path heuristic (e. g. findPath [6]).

In *Step 2a*, we compute the gradient basins and rates for the monomer landscape from the list of input structures using barriers [7] (with minh heuristic). For *Step 2b*, a list of all input structures that contain the binding pocket is generated with RNAsubopt’s constraint folding mode. This enables one to enumerate dimer structures up to a higher energy than possible for the entire landscape, ensuring the dimer world is connected. As shown in Lemma 1, the transition rates in the constrained dimer landscape are independent of the ligand’s binding energy and thus can be computed exactly like those of the monomer landscape.

In *Step 3*, the transition rates between monomer and dimer macrostates are computed based on Lemma 3 using the mapping of the monomer and dimer structures to their respective basins. For this purpose we modified barriers to output this information.

*Step 4* yields the full rate matrix *R*(*l*_0_) for one set of pre-exponential factors and a certain ligand concentration *l*_0_ by combining the previously computed rate constants. We emphasize that we can easily compute *R*(*l*_0_) for different values of *l*_0_, *A*_a_ and *A*_R_ without repeating the previous, more time-consuming computation steps.

Finally, in *Step 5* the system of ODEs is solved directly using the closed form $\vec c(t) = \exp (tR(l_{0}))\cdot \vec c(0)$, where $\vec c(t)$ is the vector of macrostate concentrations at time *t*. The exponential exp(*t* *R*(*l*_0_)) is obtained by diagonalizing *R*(*l*_0_) numerically using the tool treekin [7], which performs this computation efficiently.

### Parameters from empirical measurements

The binding energy *θ* can be derived from an empirically measured dissociation constant ${K}_{\mathrm {d}}^{\mathrm {A}}$ of the aptamer; e. g. in the case of theophylline, [18] measure a ${K}_{\mathrm {d}}^{\mathrm {A}}$ of 0.32 *μ* *M* for the theophylline aptamer of *R* *S*3. From the macroscopic measurement, we derive the binding energy as

$$\theta= RT_{\mathrm{A}} \ln \left({K}_{\mathrm{d}}^{\mathrm{A}} \cdot \text{Pr}[\,\text{``pocket''}\mid \mathrm{A}, T_{\mathrm{A}} \,]\right), $$

where *T*_A_=298*K* is the temperature of the measurement, *R* is the gas constant, and Pr[ “pocket”∣A,*T*_A_ ] denotes the equilibrium probability of the binding pocket in the aptamer at temperature *T*_A_ as calculated in the Turner energy model (cf. [1], which neglect the probability). This relation allows calculating the effective dissociation constant at temperature *T*_R_ of a theophylline riboswitch like RS3 that contains the aptamer, due to the inverse relation

$$\begin{array}{*{20}l} K^{\text{RS}}_{\mathrm{d}} &= \frac{\exp\left(\frac{\theta}{RT_{\mathrm{R}}}\right)}{\Pr[\,\text{``pocket''}\mid\text{RS},T_{\mathrm{R}}\,]} \\ &= \frac{\left({K}_{\mathrm{d}}^{\mathrm{A}} \cdot\text{Pr}[\,\text{``pocket''}\mid\mathrm{A},T_{\mathrm{A}}\,]\right)^{\frac{T_{\mathrm{A}}}{T_{\mathrm{R}}}}} {\text{Pr}[\,\text{``pocket''}\mid\text{RS},T_{\mathrm{R}}\,]}. \end{array} $$

For RS3 at *T*_R_=313.15 K,

$$ {\displaystyle \begin{array}{ll}\Pr \left[{\kern0.3em }^{``}{\mathrm{pocket}}^{"}\mid \mathrm{A},{T}_{\mathrm{A}}\kern0.3em \right]& \approx 0.292\kern2em \\ {\mathrm{and}}\\ {}\kern2em \\ {}\Pr \left[{\kern0.3em }^{``}{\mathrm{pocket}}^{"}\mid \mathrm{RS},{T}_{\mathrm{R}}\right]& \approx 2.59 \cdot p 1{0}^{-11}   \end{array}} $$

due to the pocket-constrained and unconstrained ensemble free energies in the Turner model. Thus,

$$ {\displaystyle \begin{array}{ll}\theta & \approx R{T}_{\mathrm{A}}\ln \left(0.292\underset{\mathrm{d}}{\overset{\mathrm{A}}{K}}\right)\approx -9.59\frac{\mathrm{kcal}}{\mathrm{mol}}\kern2em \\ {}{\mathrm{and}}\\ {}\kern2em \\ {}{K}_{\mathrm{d}}^{\mathrm{R}\mathrm{S}3}& \approx \frac{{\left(0.292{K}_{\mathrm{d}}^{\mathrm{A}}\right)}^{\frac{T_{\mathrm{A}}}{T_{\mathrm{R}}}}}{2.59\cdotp 1{0}^{-11}}\approx 7891\kern0.3em \mathrm{M}.\end{array}} $$

For relating the rates of the different reaction types, one needs to estimate the pre-exponential factors of all reactions. Commonly, one assumes constant factors for each type of reaction. In reasonable approximation, we furthermore equate the factors for monomer and dimer conformation changes.

Given the apparent association rate $A^{\mathrm {m}}_{\mathrm {a}}$ (which we assume to equal the macroscopic pre-exponential factor of dimerization), one can bound the microscopic pre-exponential factor *A*_a_. If we assume that refolding is much slower than dimerization, then $A^{\mathrm {m}}_{\mathrm {a}}$ is a product of the microrate and the equilibrium probability of the binding pocket. Conversely, if we assume the refolding to be much faster, than $A^{\mathrm {m}}_{\mathrm {a}}$ directly measures the dimerization microrate. Thus,

$$ A^{\mathrm{m}}_{\mathrm{a}} \; \leq \; A_{\mathrm{a}} \; \leq \; A^{\mathrm{m}}_{\mathrm{a}} \cdot \Pr[\,\text{``pocket''}\mid\text{aptamer}\,]^{-1}. $$

In the case of theophylline,

$$\text{Pr}[\,\text{``binding pocket''}\mid\text{aptamer}\,]\approx 1 $$

and consequently, *A*_a_≈*A*am.

Finally, the pre-exponential factor for dissociation *A*_d_ equals *A*_a_. This is a consequence of detailed balance of the dimerization reaction, i. e.

$$ k({R_{i} \rightarrow LR_{i}}) \,\text{Pr}[\,R_{i}\,] = k({LR_{i} \rightarrow R_{i}})\,\text{Pr}[\,LR_{i}\,], $$

which implies

$$\begin{array}{*{20}l} A_{\mathrm{a}} \,\text{Pr}[\,R_{i}\,] &= A_{\mathrm{d}} \exp(\mathfrak{b}\theta) \text{Pr}[\,R_{i}\,]\exp(-\mathfrak{b}\theta) \\ &=A_{\mathrm{d}}\,\text{Pr}[\,R_{i}\,]. \end{array} $$

### Empirical results

**Fig. 2:**
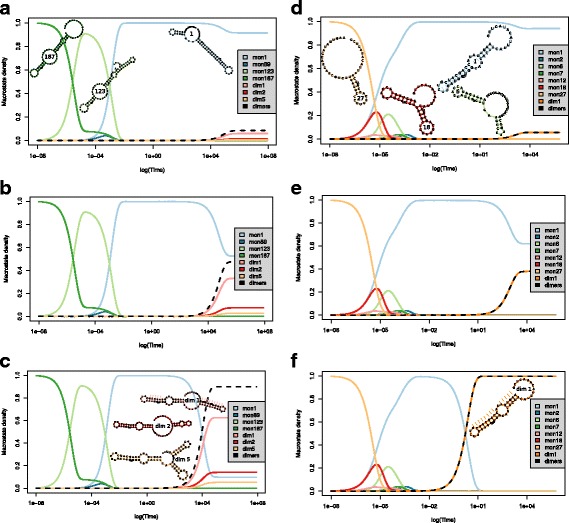
Kinetics plots showing the probabilities of prominent monomer and dimer states (*y-axis*) over time in seconds (*x-axis*) at a RNA folding rate of 10^6^ *s* ^−1^ and a dimerization rate of 600M^−1^ *s* ^−1^. Additionally, we visualize the the most prominent macrostates by their local minimum structures, which enables tracking of their coarse-grained refolding. **a–c** Complete riboswitch RS3 at concentrations 10^4^ M (**a**), 10^5^ M (**b**), and 10^5^ M (**c**). **d–f** Partially transcribed riboswitch RS3 (without 3’-half of terminator stem) at concentrations 10^−7^ M (**d**), 10^−6^ M (**e**), and 10^−3^ M (**f**). Note that since subfigures **a–c** are based on exactly the same landscapes, they share the same macrostates (e. g., mon1 in **a** and mon1 in **b** are equal). As well, this holds among subfigures **d–f**. However, across the two groups of subfigures, macrostates are not comparable (e. g., mon1 of **a** ≠ mon1 of **d**), since the landscapes differ

We apply our system to demonstrate the effect of changes in ligand concentrations to the interaction of the designed ON-switch RS3 from [1] with the ligand theophylline. Using our prototypical software, we precompute the macroprocess for RS3 including rate constants in several hours. As noted above, this yields 1133 macrostates in total, 22 of which are dimer states representing RNA molecules bound to the ligand. From the technical point of view, our system is described by 1133 coupled differential equations for the states *α*_1_,…,*α*_1111_ through *β*_1_,…,*β*_22_. Subsequently, we compute kinetics for each combination of concentrations and pre-exponential factors within seconds (on a Core i5-750 @ 4×2.67GHz). Figure 2 summarizes our results; each subfigure plots the probabilities of prominent monomer and dimer states over time. In addition, the minimum energy secondary structures is shown for the most important macrostates. It serves as a suitable representative of the macrostate’s ensemble of structures. In this sense it provides a useful, coarse-grained picture of the most likely refolding paths. We set the pre-exponential factors to the estimations *A*_R_=10^6^s^−1^ and *A*_a_=600 M^−1^s^−1^ as described before. This allows interpreting the time and ligand concentrations in concrete units and relates the speed of folding and dimerization.

Figure 2 a–c show the results for ligand concentrations 10^4^ M, 10^5^ M, and 10^6^ M. In the RS3 riboswitch, the aptamer domain is fused to a rho-independent terminator at the 3’-end. Thus, during transcription the aptamer is available shortly before the strong terminator stem can be formed and then dominates the entire structure ensemble. Therefore, we study a partially transcribed riboswitch RS3 that is shortened by the 3’-half of the terminator stem and the 3’ poly-U stretch. The kinetics of the shortened riboswitch are shown for concentrations of 10^−7^ M, 10^−6^ M, and 10^−3^M in respective Fig. 2 d–f. Note that the time scales for interaction of RS3 with theophylline are in accordance with the computed dissociation constant $K^{\text {RS3}}_{\mathrm {d}}$, which implies that the monomer and dimer concentrations are balanced at about 10^4^M ligand concentration. This extreme concentration suggests that the riboswitch would be non-functional without further, probably co-transcriptional, effects. This is a plausible hypothesis since RS3 was designed to regulate at the transcriptional level.

The estimated rates are derived from a small number of empirical measurements at different conditions, such as ion concentrations (100 mM NaCl in [12], 5 mM MgC*l*_2_ and 0.5 M NaCl in [18], no M*g* ^2+^ and 100 mM NaCl in [17]), temperatures, and actual sequences; hence they are not directly comparable. Nevertheless, they provide reasonable ball park estimates, because we observed that the qualitative behavior of the system is robust against variations of these parameters by several orders of magnitude.

## Conclusions

Several refinements of the model remain for future research. Most importantly, the assumption of only a single binding motif is rather stringent. In general, one would like to support multiple binding sites with different binding energies. Our model can be naturally generalized to such scenarios by introducing multiple “dimer worlds” corresponding to different binding motifs. Furthermore, some ligands, such as M*g* ^2+^ have multiple binding sites. The current implementation of the Arrhenius approximation of the RNA folding kinetics, finally, is quite simplistic, using only a single kinetic prefactor for all structural rearrangements. A refined model would presumably distinguishing constants for nucleation, stack extension, base pair sliding, and loop pinching. Moreover, in particular transcriptional riboswitches, which operate temporally coupled with the progressive transcription of the RNA, will be influenced by this kinetic interplay. It is well known that RNA chains commonly change their optimal structure while growing during transcription. Consequently, the RNAs refold during the process of transcription [8]. The framework presented here can be extended to co-transcriptional interaction analysis. However, this will require additional experimental measurements to calibrate the parameters of the model to properly relate the different “reaction” speeds. In particular, this entails accurate measurements of the thermodynamic parameters for the ligand binding and of the kinetic prefactors of folding and dimerization as well as the speed of transcription.

## Abbreviations

ODE: Ordinary differential equation
